# First evidence of AXL expression on circulating tumor cells in metastatic breast cancer patients: A proof‐of‐concept study

**DOI:** 10.1002/cam4.6843

**Published:** 2023-12-22

**Authors:** Thomas Bardol, Zahra Eslami‐S, Doryan Masmoudi, Marie Alexandre, Marie Duboys de Labarre, Angélique Bobrie, Véronique D'Hondt, Séverine Guiu, Keerthi Kurma, Laure Cayrefourcq, William Jacot, Catherine Alix‐Panabières

**Affiliations:** ^1^ Laboratory of Rare Circulating Human Cells—University Medical Center of Montpellier Montpellier France; ^2^ CREEC/CANECEV, MIVEGEC (CREES) Université de Montpellier, CNRS, IRD Montpellier France; ^3^ European Liquid Biopsy Society (ELBS) Hamburg Germany; ^4^ Department of Medical Oncology Institut du Cancer de Montpellier, Montpellier University Montpellier France; ^5^ Institut de Recherche en Cancérologie de Montpellier INSERM U1194, Montpellier University Montpellier France

**Keywords:** AXL, circulating biomarker, circulating tumor cells, liquid biopsy, metastatic breast cancer

## Abstract

**Background:**

For several years, the AXL tyrosine kinase receptor, a member of the Tyro3‐Axl‐Mer (TAM) family, has been considered a new strategic target in oncology. AXL overexpression is common in solid tumors and is associated with poor prognosis. In this context, the detection of a subset of circulating tumor cells (CTCs) that express AXL (AXL^+^ CTCs) could be clinically relevant.

**Methods:**

Immunostaining was performed to assess AXL expression in human breast cancer cell lines. The optimal conditions were established using flow cytometry. Spiking experiments were carried out to optimize the parameters of the CellSearch^®^ system detection test. CTC enumeration and AXL expression were evaluated in patients with metastatic breast cancer (mBC) before treatment initiation.

**Results:**

An innovative AXL^+^ CTC detection assay to be used with the CellSearch^®^ system was developed. In a prospective longitudinal clinical trial, blood samples from 60 patients with untreated mBC were analyzed to detect AXL^+^ CTCs with this new assay. CTCs were detected in 35/60 patients (58.3%) and AXL^+^ CTCs were identified in 7 of these 35 patients (11.7% of all patients).

**Conclusion:**

This newly established AXL^+^ CTC assay is a promising tool that can be used for *liquid biopsy* in future clinical trials to stratify and monitor patients with cancer receiving anti‐AXL therapies.

## Abstract 2

This newly established AXL+ CTC assay is a promising tool that could be helpful for *liquid biopsy* in future clinical trials to stratify and monitor patients with metastatic breast cancer.

## 1 INTRODUCTION

Cancer is one of the main causes of death worldwide.^1^ As it is more likely to be curable in the early stages, its early detection can boost the chances of successful treatment, resulting in fewer cancer deaths. Furthermore, cancer monitoring is critical for real‐time clinical follow‐up and for determining the minimal residual disease.^2^

*Liquid biopsy* has received considerable attention in the last decade. This term was introduced for the first time in 2010 and defines a repeatable minimal invasive procedure to detect and analyze circulating tumor cells (CTCs) in the bloodstream.^3^ More recently, the definition has been extended to other tumor‐derived circulating analytes (e.g., circulating tumor DNA, extracellular vesicles, and tumor‐educated platelets) and immune cells in the blood and also in all body fluids.^4^

After the formation and growth of the primary tumor, some more aggressive cancer cells actively detach and travel through the circulating compartment, where they are called CTCs, to reach distant organs and potentially form micrometastases.^3^ Although thousands of CTCs enter the bloodstream from the primary tumor, the metastatic process is very inefficient.^5^ Indeed, once released from the immunosuppressive tumor microenvironment, many tumor cells are likely to be attacked by immune cells. Moreover, CTCs face shear stress that strongly affects their survival.^6^ However, CTCs can escape the immune system by undergoing significant changes, such as epithelial‐to‐mesenchymal transition (EMT),^7^ or by being protected by platelets.^8^ Moreover, the CTC microenvironment supports their immune surveillance evasion and promotes their dissemination.^9^ Therefore, due to their remarkable potential as a main driver of metastasis, CTC functional and molecular characteristics might provide in‐depth knowledge on tumors and might predict the presence of tumor dissemination before clinical or radiographical signs. CTC analysis can also contribute to monitor cancer evolution. Indeed, CTC mutation profile and heterogeneity can be seen as a snapshot of cancer progression at a given time.^10^

Additionally, the expression of different markers on CTCs can predict the response to therapy. For instance, programmed death‐ligand 1 (PD‐L1) expression by CTCs might predict the response to immune checkpoint inhibitors.^11^ Estrogen receptor and epidermal growth factor receptor 2 (HER2) status in CTCs can be assessed to guide the treatment choice between chemotherapy and hormone therapy, and to refine the prognosis.^12^, ^13^ Therefore, detecting specific proteins on CTCs could help oncologists to select the most appropriate treatment for a patient in the framework of precision medicine.^4^ For several years, the AXL tyrosine kinase receptor has been considered a new strategic target in oncology.^14^, ^15^ AXL is a member of the Tyro3‐Axl‐Mer (TAM) receptor tyrosine kinase subfamily. AXL can be activated by its ligand, growth arrest‐specific‐6 (GAS‐6), or by other receptors (e.g., EGFR),^16^ and also through oxidative stress.^17^ Activation of the GAS‐6/AXL signaling pathway triggers several effector pathways, such as the RAS/RAF/MEK/ERK and PI3K/AKT cascades, that are associated with tumor growth and survival, metastatic cascade, and EMT.^18^, ^19^, ^20^, ^21^ AXL is frequently overexpressed in solid tumors, such as pancreatic and breast cancers, and is associated with worse prognosis and resistance to antitumor treatments (radiotherapy, chemotherapy, and targeted therapy).^19^, ^21^, ^22^, ^23^ AXL is also involved in modifying the tumor microenvironment^24^ and in hijacking the immune system.^25^ For example, AXL overexpression at the cancer cell surface correlates with decreased expression of the major histocompatibility complex 1 (MHC‐1) and increased expression of the immune checkpoint PD‐L1, leading to decreased CD8^+^ T‐cell antitumor activity.^26^, ^27^ AXL signaling also promotes a pro‐tumorigenic microenvironment through the secretion of cytokines that alter macrophage polarization and lymphocyte infiltration.^28^, ^29^

AXL clinical relevance and oncogenic potential have been largely described,^22^, ^23^, ^30^ especially in breast cancer. Indeed, AXL is overexpressed in 50%–75% of breast cancer samples. This overexpression has been correlated with clinical features of tumor aggressiveness and poor prognosis.^19^, ^21^, ^22^, ^31^ However, no data are available on AXL expression in CTCs from patients with breast cancer. Therefore, to determine whether CTCs express AXL, we optimized an AXL^+^ CTC detection assay to be used with the CellSearch^®^ system and analyzed blood samples from patients with metastatic breast cancer (mBC) before treatment. We could detect a CTC subset that expresses AXL and that might be used to stratify patients eligible to anti‐AXL targeted therapies.

## 2 METHODS

### 2.1 Cell culture

The two breast cancer cell lines MDA‐MB‐231 (ATCC^®^ CRM HTB‐26™, RRID:CVCL_062e) and SK‐BR‐3 (ATCC^®^ HTB‐30™, RRID:CVCL_0033) were used to optimize the assay for AXL^+^ CTC detection with the CellSearch^®^ system. Both cancer cell lines were derived from patients with mBC.^32^ They were cultured in 25‐cm^2^ adherent flasks in Dulbecco's modified Eagle medium‐high glucose (Dominic Deutscher, ref: L0103‐500) supplemented with 10% fetal bovine serum (FBS) (Eurobio, ref: CVFSVF00‐01), as recommended by ATCC^®^. Cells were maintained in a humidified atmosphere with 5% CO_2_ at 37°C.

### 2.2 Immunofluorescence staining

To determine which anti‐human AXL antibody to use in our assay, AXL expression in MDA‐MB‐231 and SK‐BR‐3 cells was analyzed by immunofluorescence (IF) with two anti‐human AXL antibodies: (i) an antibody (R and D Systems Cat# MAB154, RRID:AB_2062558) manually coupled to Alexa Fluor^®^ 555 (Alexa Fluor^®^ 555 Antibody Labeling Kit, ref: A20187) at the final concentration of 5 μg/mL and (ii) an anti‐human AXL‐phycoerythrin (PE) antibody (R and D Systems Cat# FAB154P, RRID:AB_2894899) at the final concentration of 0.5 μg/mL. The SK‐BR‐3 cell line that does not express AXL was used as negative control.^33^ Cells were gently centrifuged and deposited on standard glass slides using a Cytospin™ 4 Cytocentrifuge (Thermo Scientific) and after staining, they were mounted in ProLong Gold antifade reagent with DAPI (Invitrogen, Ref: P36931). Staining was observed under a fluorescence microscope (Axio Imager M1, Carl Zeiss Vision, Hallbergmoos, Germany) and analyzed with the Zen Blue Edition software. Cells were also stained with an anti‐human EpCAM^FITC^ antibody (Miltenyi Biotec Cat# 130–113‐263, RRID:AB_2726064), at the final concentration of 7.5 μg/mL. All antibodies were incubated at room temperature for 30 min.

### 2.3 Flow cytometry analyses

The best antibody (R and D Systems Cat# FAB154P, RRID:AB_2894899), at the final concentration of 0.5 μg/mL, was tested also by flow cytometry. A solution of phosphate‐buffered saline (PBS) supplemented with 2% FBS and 2 mM ethylenediaminetetraacetic acid (EDTA) was used for cell staining and washing. MDA‐MB‐231 and SK‐BR‐3 cells were collected and stained with the FAB‐154P antibody or the corresponding isotypic control (Miltenyi Biotec Cat# 130–119‐684, RRID:AB_2751802) on ice for 30 min. Staining was then analyzed using a Gallios flow cytometer (Beckman Coulter, Brea, CA, USA) and the Kaluza software at the IRMB Montpellier Rio‐Imaging Cytometry Core.

### 2.4 Patient samples, blood collection, and ethics

Blood samples were obtained from patients included in the basket clinical trial ALCINA 2 (ClinicalTrials.gov NCT04025541; registered on July 19, 2019). For this trial, ≥18‐year‐old patients with histologically confirmed stage IV breast cancer were prospectively recruited before initiation of their first treatment for mBC at the Montpellier Cancer Institute (ICM), between April 2022 and April 2023. All patients provided their written informed consent and were prospectively included. The basket clinical trial ALCINA 2 was approved by the local ethics committee (CPP Ouest 4). The study protocol can be found on ClinicalTrials.gov. Their baseline characteristics were extracted from their electronic health record. The biospecimen reporting for improved study quality (BRISQ) criteria are in Table 1.

**TABLE 1:** Biospecimen reporting for improved study quality (BRISQ) checklist.

Data elements	
Biospecimen type	Whole blood
Site of blood draw	Antecubital area of the arm (peripheral blood)
Clinical diagnosis of patients	Breast cancer
Disease status of patients	Metastatic breast cancer
Clinical characteristics of patients	Patients with breast cancer eligible for first‐line chemotherapy
Vital status of patients	Alive
Pathology diagnosis	Intraductal carcinoma or lobular carcinoma Evaluation of estrogen, progesterone receptors, and HER2 level of expression
Collection mechanism	Pre‐chemotherapy blood draw
Type of stabilization	CellSave tubes
Type of long‐term preservation	Fixation at room temperature (25°C)
Constitution of preservative	CellSave tube (Menarini patent)
Storage temperature	Room temperature (25°C)
Storage duration	Up to 72 h
Shipping temperature	Room temperature (25°C)
Composition assessment and selection	Presence of at least one circulating tumor cell AXL expression on the cell surface

*Note*: Preanalytical variables are addressed in this BRISQ list.

Abbreviation: HER2, human epidermal growth factor receptor 2.

At inclusion, blood samples were collected in 10‐mL CellSave^®^ tubes (Menarini Silicon Biosystems, ref: 7900005). Tubes were stored at room temperature and sent to the Laboratory of Rare Human Circulating Cells (LCCRH), Montpellier University Hospital, Montpellier, France, for processing within 72 h.

For the spiking experiments, blood samples from healthy donors were collected in 6 mL purple‐cap BD vacutainers containing the anti‐coagulant EDTA (BD‐Plymouth ref: 367864A) at the Établissement Français du Sang (EFS) and processed at the University Medical Center of Montpellier, France. All donors gave their consent for blood collection for research purpose.

### 2.5 CTC detection

For CTC detection, the Food & Drugs Agency (FDA)‐cleared CellSearch^®^ system (Menarini Silicon Biosystems, Inc.; Bologna, Italy) was used. Blood samples were processed with the CellSearch^®^ CXC kit (Menarini Silicon Biosystems, ref: 7900017) and the Celltracks^®^ Autoprep^®^ System (Menarini Silicon Biosystems, Inc.; Bologna, Italy), according to the manufacturer's instructions. Briefly, using 7.5 mL of each blood sample, EpCAM^+^ CTCs were enriched and captured (positive‐enrichment method), followed by CTC detection using anti‐cytokeratin (CK)‐fluorescein isothiocyanate‐conjugate (CK8, 18 and 19) and anti‐CD45 allophycocyanin (to exclude leukocytes) antibodies, and nuclear staining with 4′‐6‐diamidino‐2‐phenylindole (DAPI). The fourth channel was available to incorporate a user‐defined PE conjugate (for AXL expression in the present study). After immunocytochemical staining, immunomagnetically labeled cells were kept in a magnetic field and scanned using the CellTracks Analyzer II^®^ (Menarini Silicon Biosystems, Inc.; Bologna, Italy). Detected elements were reviewed by a certified technician and CTC identification was confirmed by an experienced biologist according to standardized criteria. Due to the exploratory design of the present study, a threshold of 1 CTC/7.5 mL was used to consider a patient sample CTC‐positive (CTC^+^).

### 2.6 Statistical analyses

Numeric variables were expressed as mean ± standard deviation (SD) and discrete outcomes as absolute and relative (%) frequencies. Patients with CTC^+^ sample were classified in two groups: with AXL^+^ CTCs and with AXL^−^ CTCs. Their similarity was assessed by comparing their baseline demographic data. Normality and heteroskedasticity of continuous data were assessed with the Shapiro–Wilk and Levene's test, respectively. Continuous outcomes were compared with the unpaired Student's *t‐*test, Welch's *t*‐test, or Mann–Whitney *U*‐test according to the data distribution. Discrete outcomes were compared with the chi‐squared or Fisher's exact test. The alpha risk was set at 5% and two‐tailed tests were used.

Statistical analyses were performed with EasyMedStat (version 3.26; www.easymedstat.com).

## 3 RESULTS

### 3.1 Development of an assay to detect AXL ^+^ CTCs with the CellSearch ^®^ system using breast cancer cell lines

**FIGURE 1:**
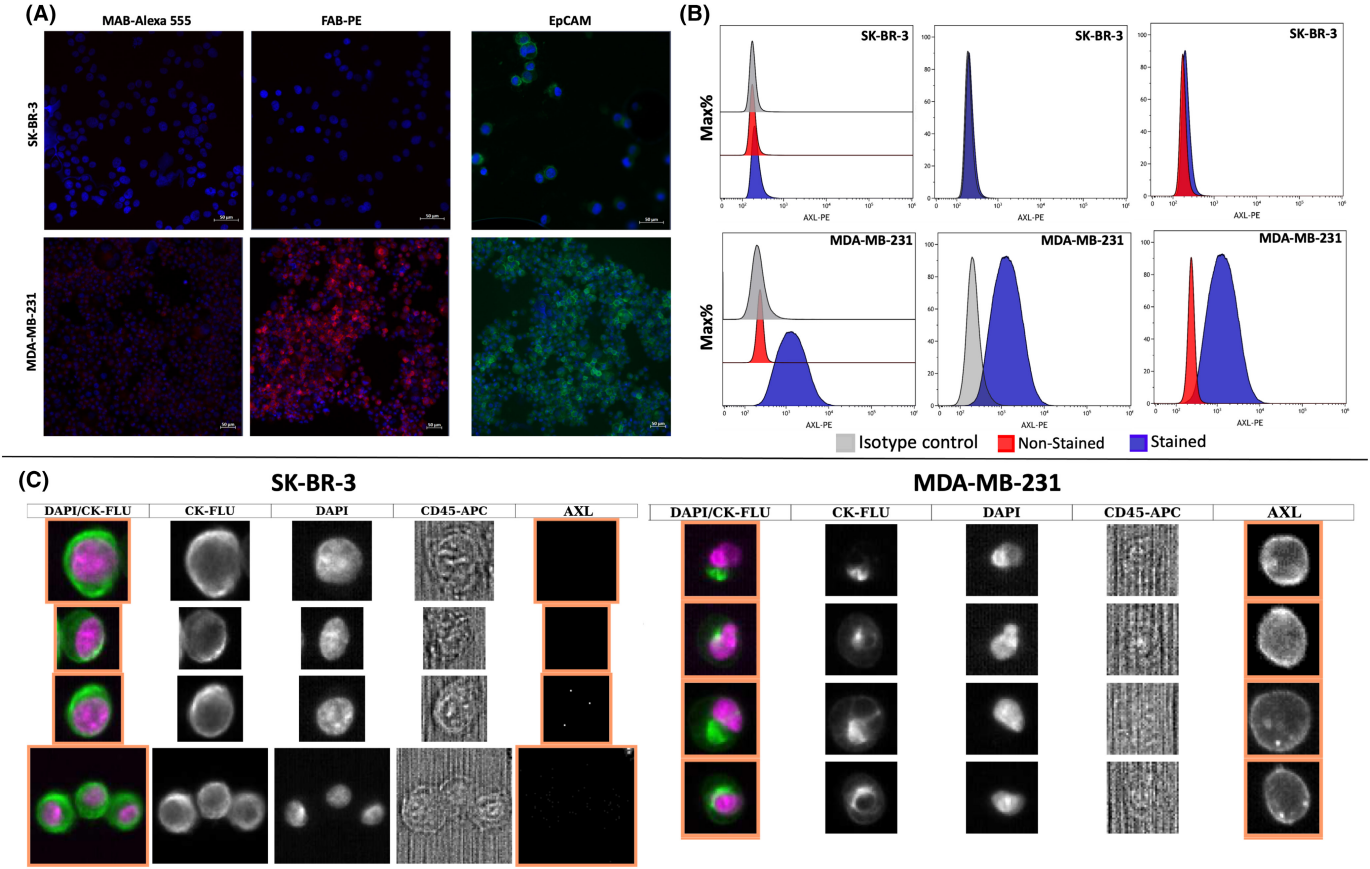
Validation of the sensitivity and specificity of the anti‐human AXL antibody FAB‐154P for the detection of AXL^+^ CTCs. (A) Immunocytochemical analyses. Representative images (20× magnification) of SK‐BR‐3 and MDA‐MB‐231 cells stained for AXL (red), epithelial cell adhesion molecule (EpCAM) (green) and DAPI (blue). Scale bars: 50 μm. (B) Flow cytometry analyses. Validation of the anti‐AXL antibody FAB‐154P (in blue) in SK‐BR‐3 and MDA‐MB‐231 cells compared with the isotypic control (IgG1) (in gray) and unstained cells (in red). (C) CellSearch^®^ system analyses. SK‐BR‐3 and MDA‐MB‐231 cells added to blood samples from healthy donors were identified with the CellSearch^®^ system using the anti‐human AXL antibody FAB‐154P in the fourth channel. All tests were performed three times and the figures depict representative results.

For developing an assay to detect single AXL^+^ CTCs, two commercially available breast cancer cell lines were used to determine the sensitivity, specificity, and dynamic range of two anti‐AXL antibodies (MAB154R antibody coupled to Alexa Fluor 555 and FAB‐154P) by IF analysis and flow cytometry, and then in the CellSearch^®^ system (Figure 1A–C).

First, AXL expression on MDA‐MB‐231 and SK‐BR‐3 cells was tested by IF. No AXL signal was detected in the SK‐BR‐3 cell line (negative control) with both antibodies. Conversely, a strong positive signal was detected on MDA‐MB‐231 cells using the FAB‐154P antibody (positive control) compared with the MAB154R antibody coupled to Alexa Fluor^®^ 555 (Figure 1A). Flow cytometry analysis of both cell lines using the selected antibody (FAB‐154P) confirmed that AXL expression could be detected in MDA‐MB‐231 cells, but not in SK‐BR‐3 cells (Figure 1B). Moreover, the absence of labeling with the PE isotype control confirmed the specificity of the FAB‐154P antibody. Analysis of epithelial cell adhesion molecule (EpCAM) expression indicated that both cancer cell lines expressed EpCAM (Figure 1A).

For the optimization step with the CellSearch^®^ system, the CXC Control Cell Kit and the FAB‐154P antibody in the fourth channel were used to analyze SK‐BR‐3, and MDA‐MB‐231 cells. One thousand cells diluted in 7.5 mL of PBS were used to select the optimal antibody concentration and exposure time. Two antibody concentrations (5 and 10 μg/mL) and three different exposure times (0.1, 0.2, and 0.3 s) were tested. Again, AXL expression was detected in MDA‐MB‐231 cells, but not in SK‐BR‐3 cells. The optimal staining was obtained with 10 μg/mL of antibody and 0.2 s of exposure time, as recommended by the CellSearch^®^ system manufacturer (Figure 1C).

### 3.2 Validation of AXL detection in the CellSearch ^®^ system using breast cancer cells spiked in blood samples of healthy donors

**FIGURE 2:**
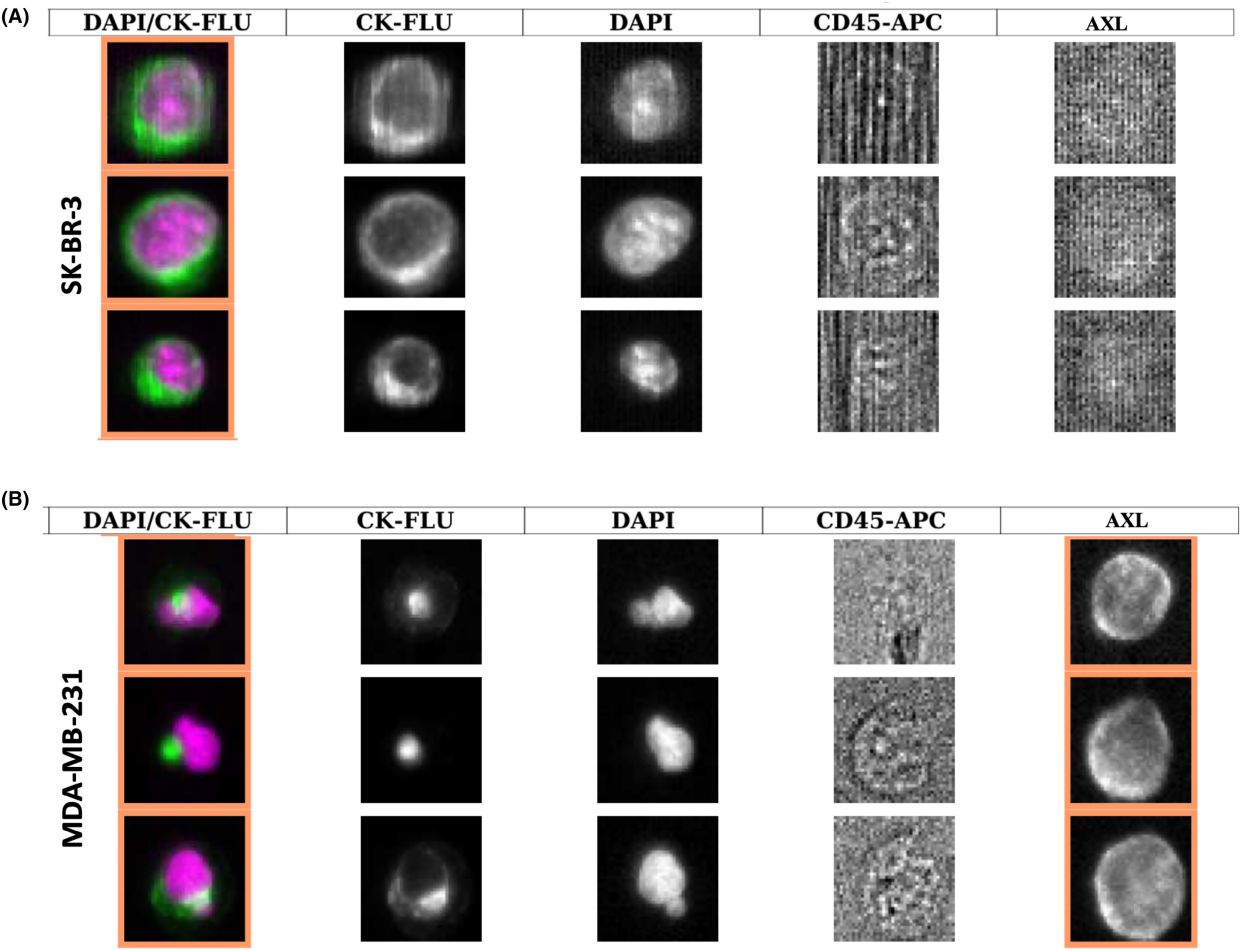
Representative images of AXL expression on (A) SK‐BR‐3 and (B) MDA‐MB‐231 cells using the CellSearch^®^ system. Column 1 represents the merge picture, which is the superposition of cytokeratin (CK) expression, shown in column 2, DAPI staining, shown in column 3, and cluster of differentiation 45 (CD45) staining, shown in column 4. AXL expression is in column 5.

To mimic CTCs in blood samples from patients with breast cancer, 7.5 mL of each blood sample from three healthy donors was spiked with 100 SK‐BR‐3 or MDA‐MB‐231 cells, and cancer cells were detected with the CellSearch^®^ system (10 μg/mL of antibody and 0.2 s of exposure time). The AXL positivity rate of the CellSearch^®^ CTC‐AXL assay was defined as the ratio between the number of AXL^+^ cancer cells detected by the CellTracks analyzer and the initial 100 cancer cells (Figure 2A,B **)**. In samples spiked with MDA‐MB‐231 cells, AXL detection achieved a sensitivity of 0.88 and a specificity of 1.00. These results validated the “CTC‐AXL” assay, using the CellSearch^®^ system, to detect AXL^+^ cancer cells in blood samples.

### 3.3 AXL status in CTCs from patients with mBC

Next, the “CTC‐AXL” assay was performed using peripheral blood samples from 60 patients with mBC before the first‐line treatment of the metastatic disease. The patients' median age at inclusion was 63.5 years. The median time from the initial breast cancer diagnosis to inclusion was 40 months (0–366). Thirty‐seven patients (62%) presented metachronous metastases (diagnosis more than 6 months after the primary tumor detection) with a median time from diagnosis to metastatic disease of 44 months. The mean number of metastatic sites was two (1–5). Ductal carcinoma was the most common histological type (*n* = 53/60, 88%). The cohort was heterogeneous regarding hormone receptors and human epidermal growth factor receptor 2 (HER2) status: Fifty patients had hormone receptor‐positive and HER2‐negative breast cancer (83.3%), nine had HER2‐positive breast cancer (15.7%), and one patient had triple negative breast cancer (2%). The patients' characteristics are described in Table 2.

**TABLE 2:** Patients' clinical and histological characteristics.

Variables	Total	CTC+	AXL^+^ CTCs	AXL^−^ CTCs	*p‐*value
Patients	60	35 (58)	7 (20)	28 (80)	
Sex					>0.999
Female	59 (98)	34 (97)	7 (100)	27 (96)	
Male	1 (2)	1 (3)	0	1 (4)	
Age (years), median (range)	63 (33–89)	59 (33–80)	63 (43–76)	58 (33–80)	0.407
Metastatic disease features					
Synchronous metastases	23 (38)	12 (34)	2 (29)	10 (36)	>0.999
Metachronous metastases	37 (62)	23 (66)	5 (71)	18 (64)	
Time from initial breast cancer diagnosis to inclusion (months), median (range)	40 (0–366)	37 (0–366)	87 (0–290)	37 (0–366)	0.481
Number of metastatic sites, median (range)	2 (1–5)	2 (1–5)	2 (1–5)	2 (1–5)	>0.999
Histological type					
Ductal carcinoma	53 (88.3)	31 (89)	5 (71)	26 (93)	0.171
Lobular carcinoma	7 (11.7)	4 (11)	2 (29)	2 (7)	
Histological (SBR) grade					
1	7 (12)	5 (14)	3 (43)	2 (7)	0.036*
2 or 3	43 (72)	27 (77)	3 (43)	24 (86)	
X	10 (16)	3 (9)	1 (14)	2 (7)	
Receptors status					
HR+/HER2−	50 (83)	29 (83)	6 (86)	22 (79)	0.843
HR+/HER2+	7 (12)	4 (11)	1 (14)	3 (11)	
HR−/HER2+	2 (3)	1 (3)	0	2 (7)	
HR−/HER2−	1 (2)	1 (3)	0	1 (3)	

*Note*: AXL + CTC and AXL−CTC groups were compared. Values are shown as numbers (percentages) unless otherwise indicated. Bold value is to underline the significance.

Abbreviations: HER2, human epidermal growth factor receptor 2; HR, hormone receptors, including estrogen receptor and progesterone receptor; SBR grade, Scarff Bloom and Richardson grading system. *Statistically significant.

**FIGURE 3:**
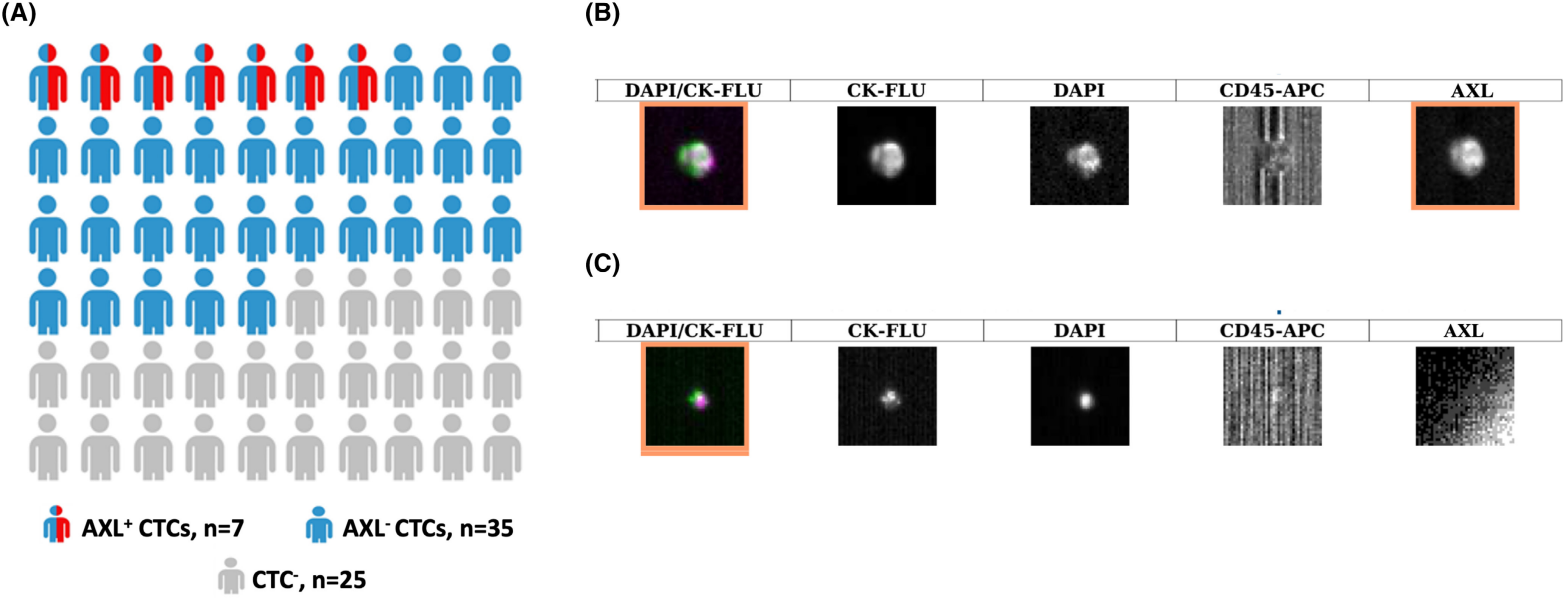
AXL expression analysis in CTCs from patients with metastatic breast cancer (mBC) detected with the CellSearch^®^ system. (A) Diagram showing the number of patients without CTCs (CTCs^−^), with CTCs (CTCs^+^) and with AXL^+^ CTCs at baseline in the ALCINA 2 cohort (*n* = 60 patients). (B,C) Identification of AXL expression on CTCs using the CellTracks Analyzer II^®^. Column 1 shows the merge picture, which is the superposition of columns 2–4. AXL expression is shown in column 5 with an orange frame. The intensity of AXL staining varied among patients and among CTCs in the same blood sample.

Using the CellSearch^®^ system, at least 1 CTC was identified in 35/60 blood samples (58.3%). CTC numbers ranged from 1 to 962. Among the 60 samples examined (1 sample per patient), seven (11.7% of the total) contained AXL^+^ CTCs (ranging from 1 to 8 cells) (Table 3). Samples with AXL^+^ CTCs represented 20% of all CTC^+^ samples (Figure 3).

**TABLE 3:** CTC enumeration and AXL expression in patients with metastatic breast cancer (mBC) and CTCs.

ID	Sex	Age	Histological type	SBR grade	Receptors status	Metastatic sites	Total CTC count	AXL^+^ CTC count
AXL S01	Female	61	Ductal	3	HR+/HER2−	2	16	0
AXL S02	Female	70	Ductal	2	HR+/HER2−	1	2	0
AXL S04	Female	71	Ductal	1	HR+/HER2−	3	**3**	**1 (33%)**
AXL S05	Female	72	Ductal	2	HR+/HER2−	2	181	0
AXL S06	Female	52	Ductal	3	HR+/HER2−	1	13	0
AXL S07	Female	52	Ductal	2	HR+/HER2+	2	1	0
AXL S09	Female	52	Ductal	1	HR+/HER2−	1	**18**	**8 (44%)**
AXL S12	Female	66	Ductal	1	HR+/HER2+	2	**14**	**1 (7%)**
AXL S14	Female	33	Ductal	2	HR+/HER2−	3	75	0
AXL S16	Female	55	Ductal	2	HR+/HER2−	1	1	0
AXL S17	Female	70	Lobular	2	HR+/HER2−	1	962	0
AXL S18	Female	67	Lobular	2	HR+/HER2−	1	1	0
AXL S20	Female	39	Ductal	3	HR+/HER2+	1	5	0
AXL S24	Female	69	Ductal	3	HR+/HER2+	1	4	0
AXL S25	Female	49	Ductal	2	HR+/HER2−	4	2	0
AXL S26	Female	71	Ductal	2	HR+/HER2−	1	2	0
AXL S28	Female	55	Ductal	2	HR+/HER2−	1	15	0
AXL S30	Female	80	Ductal	3	HR+/HER2−	1	2	0
AXL S31	Female	59	Ductal	2	HR+/HER2−	2	1	0
AXL S32	Male	76	Ductal	3	HR+/HER2−	2	2	0
AXL S34	Female	43	Ductal	2	HR+/HER2−	1	**138**	**5 (4%)**
AXL S38	Female	57	Ductal	1	HR+/HER2−	2	12	0
AXL S39	Female	46	Ductal	2	HR+/HER2−	3	3	0
AXL S40	Female	34	Ductal	X	HR‐/HER2−	5	1	0
AXL S41	Female	37	Ductal	3	HR+/HER2−	2	189	0
AXL S44	Female	76	Ductal	1	HR+/HER2−	2	8	0
AXL S45	Female	70	Ductal	3	HR+/HER2−	5	**9**	**1 (11%)**
AXL S46	Female	65	Ductal	2	HR/HER2+	2	7	0
AXL S47	Female	50	Ductal	2	HR+/HER2−	4	66	0
AXL S52	Female	63	Lobular	X	HR+/HER2−	3	**47**	**3 (6%)**
AXL S53	Female	59	Ductal	2	HR+/HER2−	1	6	0
AXL S56	Female	76	Lobular	2	HR+/HER2−	1	**22**	**1 (5%)**
AXL S58	Female	57	Ductal	2	HR+/HER2−	2	5	0
AXL S59	Female	40	Ductal	X	HR+/HER2−	1	4	0
AXL S61	Female	79	Ductal	2	HR+/HER2−	1	19	0

*Note*: CTCs were detected using the CellSearch^®^ CXC KIT and AXL staining in the fourth channel. Values are shown as numbers (percentages) unless otherwise indicated. Bold values are to highlight the patients that are CTC‐AXL+.

Abbreviations: HER2, human epidermal growth factor receptor 2; HR, Hormone Receptors, including estrogen receptor and progesterone receptor; SBR grade, Scarff Bloom and Richardson grading system.

Lastly, to assess potential associations between the presence of AXL^+^ CTCs and the baseline clinical and pathological features, patients with CTCs were divided in two groups: with AXL^+^ CTCs (*n* = 7) and with AXL^−^ CTCs (*n* = 28). The groups were similar in terms of baseline demographic data (all *p*‐values > 0.05). There was no correlation between AXL^+^ CTC detection and metastatic disease features, histological type, and receptor status (all *p*‐values > 0.05). AXL^+^ CTC detection was only associated with the tumor histological grade (*p* = 0.036).

## 4 DISCUSSION

The most aggressive cancer cells can detach from the primary tumor, travel through the bloodstream as CTCs, and reach a distant site where they begin to colonize a new distant tissue, and form metastases.^3^ Personalized medicine is based on the comprehensive evaluation of many prognostic and/or predictive biomarkers (e.g., PD‐L1 expression is associated with the risk of metastasis).^34^ Therefore, it seems relevant to investigate the presence of different biomarkers to refine patient stratification.

Here, we described an assay to detect AXL expression on the surface of CTCs from patients with mBC using the CellSearch^®^ system, the only FDA‐approved method to detect CTCs in metastatic breast, prostate, and colon cancer.^35^, ^36^, ^37^ We found that the detection of AXL^+^ CTCs was associated with the tumor histological (SBR) grade. This is consistent with previous studies that did not find any clear correlation between AXL expression and clinicopathological features.^38^, ^39^ Detection of AXL^+^ CTCs was reported in lung cancer using an automated micro‐cavity array system.^40^ The authors found that AXL^+^ CTCs were more frequently detected among vimentin‐positive CTCs than CK‐positive CTCs. This indicates that mesenchymal markers must be incorporated for better detection of AXL^+^ CTCs in lung cancer. Moreover, vimentin^+^ AXL^+^ CTC detection significantly correlated with the number of distant metastatic sites.^40^ However, the authors stated that this assay was specifically developed for capturing CTCs in blood samples from patients with lung cancer due to the low efficiency of the CellSearch^®^ system for this cancer.

Optimization steps allowed us to develop an efficient assay that is ready to use for translational clinical trials. We detected CTCs and AXL^+^ CTCs in 58.3% and 11.7% patients of our cohort, respectively. The expected CTC detection rate (≥1 CTCs) for mBC is ~70% with the CellSearch^®^ Epithelial Cell Kit.^41^ In our study, the overall CTC detection rate was slightly lower compared with previously reported studies. This might be explained by the high percentage of patients with HER2‐negative mBC (85%) in our cohort. Indeed, a recent study in a very large cohort of patients with HER2‐negative mBC (*n* = 1933) found a CTC detection rate of 63%.^13^

Moreover, AXL^+^ CTC detection might have been influenced by the technology used to identify/enumerate CTCs. Indeed, as AXL is a well‐known EMT driver, AXL overexpression in human mammary cells leads to downregulation of epithelial markers and upregulation of mesenchymal markers. However, even when CTCs have started the EMT, they still express EpCAM at the surface.^42^ Therefore, the CellSearch^®^ system, which is an EpCAM‐based method for CTC detection and the only FDA‐cleared system, is still a robust and reproducible technology in mBC. Moreover, it offers the possibility to add an additional marker in the last channel to characterize CTCs. However, as only EpCAM^+^ CTCs were selected and analyzed, the real number of AXL^+^ CTCs might be even higher. The CellSearch^®^ system is the only automated technical platform with remarkable reproducibility, but it is imperative to explore and develop new methods to address this limitation.^2^, ^4^

In our cohort, AXL^+^ CTCs were detected in 11.7% of samples. This is lower than the rate of AXL tissue expression reported in breast cancer, which can reach 57.6%.^33^ Another study also reported similar percentage of AXL tissue expression (56.67%), indicating a significant association between malignancy and high AXL expression.^39^ Moreover, AXL overexpression leads to therapeutic resistance, making AXL^+^ CTC detection a candidate biomarker to predict treatment response.^21^ Indeed, AXL inhibitors are considered a promising therapeutic strategy in mBC.^43^, ^44^ Specific and non‐specific AXL and kinase inhibitors have been or are currently tested in several trials (Table S1 lists clinical trials on agents targeting AXL or its ligand GAS6). Results showed that combining conventional therapies, such as chemotherapy, with AXL‐targeting drugs increases the effectiveness of treatment, making this receptor a rising theranostic biomarker.^45^ Therefore, monitoring AXL expression on the surface of cancer cells could become a companion biomarker of AXL targeted therapy efficacy.

In this proof‐of concept study to determine whether AXL^+^ CTC can be detected using the only FDA‐approved CTC detection system, a CTC cut‐off of one was used to define positivity. A statistically defined CTC cut‐off will be used to analyze the prognostic impact of AXL^+^ CTC detection at baseline after the 36‐month follow‐up.

## 5 CONCLUSION

We reported for the first time the detection of AXL+ CTCs with the CellSearch^®^ system in 60 patients with mBC. The clinical impact of their detection will be assessed after a longer follow‐up.

## AUTHOR CONTRIBUTIONS

**Thomas Bardol:** Conceptualization (equal); formal analysis (equal); investigation (equal); writing – original draft (equal); writing – review and editing (equal). **Zahra Eslami‐S:** Conceptualization (equal); formal analysis (equal); investigation (equal); writing – original draft (equal); writing – review and editing (equal). **Doryan Masmoudi:** Conceptualization (equal); formal analysis (equal); investigation (equal); writing – original draft (equal); writing – review and editing (equal). **Marie Alexandre:** Resources (equal); writing – review and editing (equal). **Marie Duboys de Labarre:** Resources (equal); writing – review and editing (equal). **Angélique Bobrie:** Resources (equal); writing – review and editing (equal). **Véronique D'Hondt:** Resources (equal); writing – review and editing (equal). **Séverine Guiu:** Resources (equal); writing – review and editing (equal). **Keerthi Kurma:** Formal analysis (equal); investigation (equal); writing – review and editing (equal). **Laure Cayrefourcq:** Formal analysis (equal); methodology (equal); writing – review and editing (equal). **William Jacot:** Resources (equal); supervision (equal); validation (equal); writing – review and editing (equal). **Catherine Alix‐Panabières:** Conceptualization (lead); supervision (equal); validation (equal); writing – review and editing (equal).

## CONFLICT OF INTEREST STATEMENT

Catherine Alix‐Panabières is one of the patent holders (US Patent Number 16,093,934) for detecting and/or characterizing circulating tumor cells. She received an honorarium from Menarini©. William Jacot is on the Astra Zeneca, Eisai, BMS, Lilly France, Daiichi Sankyo, MSD, Novartis, Pfizer, Roche, and Seagen advisory boards and received honoraria or payment from Astra Zeneca, Eisai, BMS, Lilly France, Daiichi Sankyo, MSD, Novartis, Pfizer, Roche and Seagen. William Jacot also received meeting travel support from Astra Zeneca, Novartis, Chugai Pharma, Pfizer, Eisai, Pierre Fabre, Glaxo Smithkline, Roche, Lilly France, Sanofi, and Aventis. The remaining authors declare no conflict of interest.

## ETHICS STATEMENT

All patients provided their written informed consent and were prospectively included in the ethics committee‐approved ALCINA 2 study.

## TRIAL REGISTRATION NUMBER

NCT04025541. Registered 19 July 2019.

## Supporting information


**Table S1:** **Ongoing clinical trials of agents targeting AXL or its ligand GAS 6** (*Source*: ClinicalTrial.gov).

## Data Availability

Availability of data and materials: All data analyzed during this study are included in this published article.
