# Supplementary material for: First evidence of AXL expression on circulating tumor cells in metastatic breast cancer patients: A proof‐of‐concept study
Source: Cancer Med. 2023 Dec 22;13(1):e6843. doi: 10.1002/cam4.6843 (PMC10807582; doi:10.1002/cam4.6843)
Supplement: Supplementary file 1 — Table S1: Ongoing clinical trials of agents targeting AXL or its ligand GAS 6 (Source: ClinicalTrial.gov). [file CAM4-13-e6843-s001.doc]

**Supplementary Table 1: Ongoing clinical trials of agents targeting AXL or its ligand GAS 6** (Source ClinicalTrial.gov)

| **Type of compound** | **Compound** | **Cancer types** | **Recruitment status** | **Clinical Trial** | **Phase** | **Date** |
| --- | --- | --- | --- | --- | --- | --- |
| ***Small AXL inhibitors*** | Sitravatinib (MGCD516) | Advanced cancer | Active | NCT02219711 | I | 2014 |
|  |  |  | Not Recruiting |  | Ib |  |
|  | Bemcentinib (BGB324) | Non-Small Cell Lung Cancer | Completed | NCT02424617 | I | 2015 |
|  | Erlotinib |  |  |  | II |  |
|  | Gilteritinib (ASP2215) | Advanced Solid Tumors | Completed | NCT02456883 | I | 2015 |
|  | BPI-9016M | Solid Tumors | Completed | NCT02478866 | I | 2015 |
|  | Glesatinib (MGCD265) | Non-Small Cell Lung Cancer | Completed | NCT02544633 | II | 2015 |
|  | Dubermatinib (TP-0903) | Advanced Solid Tumors | Active | NCT02729298 | I | 2016 |
|  |  | EGFR Positive Non-small Cell Lung Cancer | Not recruiting |  |  |  |
|  |  | Colorectal Carcinoma |  |  |  |  |
|  |  | Recurrent Ovarian Carcinoma |  |  |  |  |
|  |  | BRAF-Mutated Melanoma |  |  |  |  |
|  | BPI-9016M | c-Met- Dysregulated Advanced Non-small Cell Lung Cancer | Recruiting | NCT02929290 | I | 2016 |
|  | Bemcentinib (BGB324) | Non-Small Cell Lung Carcinoma | Active | NCT02922777 | I | 2016 |
|  |  |  | Not recruited |  |  |  |
|  | Dubermatinib (TP-0903) | Advanced Solid Tumors | Active | NCT02729298 | I | 2016 |
|  |  | EGFR Positive Non-small Cell Lung Cancer | Not recruiting |  |  |  |
|  |  | Colorectal Carcinoma |  |  |  |  |
|  |  | Recurrent Ovarian Carcinoma |  |  |  |  |
|  |  | BRAF-Mutated Melanoma |  |  |  |  |
|  | Bemcentinib (BGB324) | Triple Negative Breast Cancer | Terminated | NCT03184558 | II | 2017 |
|  |  | Inflammatory Breast Cancer Stage IV |  |  |  |  |
|  | ONO-7475 | Acute Leukemia | Recruiting | NCT03176277 | I | 2017 |
|  |  | Myelodysplastic Syndromes |  |  | II |  |
|  | INCB081776 | Advanced Solid Tumors | Recruiting | NCT03522142 | I | 2018 |
|  | INCMGA00012 |  |  |  |  |  |
|  | SLC-391 | Solid Tumor | Recruiting | NCT03990454 | I | 2019 |
|  | Bemcentinib (BGB324) | Acute Myeloid Leukemia | Completed | NCT03824080 | II | 2019 |
|  |  | High-risk Myelodysplastic Syndrome |  |  |  |  |
|  |  | Low-risk Myelodysplastic Syndrome |  |  |  |  |
|  | Bemcentinib (BGB324) | Brain and Central Nervous System Tumors | Active | NCT03965494 | I | 2019 |
|  |  |  | Not recruiting |  |  |  |
|  | SLC-391 | Solid Tumor | Recruiting | NCT03990454 | I | 2019 |
|  | PF-07265807 | Neoplasm Metastasis | Recruiting | NCT04458259 | I | 2020 |
|  | Q702 | Solid Tumor | Recruiting | NCT04648254 | I | 2020 |
|  |  | Advanced Cancer |  |  |  |  |
|  |  | Metastatic Cancer |  |  |  |  |
| ***Small AXL inhibitors combined with small EGFR inhibitors*** | DS-1205c | Non-Small Cell Lung Cancer | Completed | NCT03599518 | I | 2018 |
|  | Gefitinib |  |  |  |  |  |
| ***Small AXL inhibitors combined with chemotherapy*** | Bemcentinib (BGB324) | Acute Myeloid Leukemia | Active | NCT02488408 | I | 2015 |
|  | Cytarabine | Myelodysplastic Syndromes | Not recruiting |  | II |  |
|  | Decitabine |  |  |  |  |  |
|  | Bemcentinib (BGB324) | Non-Small Cell Lung Carcinoma | Active | NCT02922777 | I | 2016 |
|  | Docetaxel |  | Not recruiting |  |  |  |
|  | Bosutinib (SKI-606) | Carcinoma, Non-Small-Cell Lung | Completed | NCT03023319 | I | 2017 |
|  | Pemetrexed | Mesothelioma |  |  |  |  |
|  |  | Bladder Cancer |  |  |  |  |
|  |  | Ovarian Cancer |  |  |  |  |
|  |  | Peritoneal Cancer |  |  |  |  |
|  |  | Thymoma |  |  |  |  |
|  |  | Thymus Cancer |  |  |  |  |
|  |  | Uterine Cervical Cancer |  |  |  |  |
| ***Small AXL inhibitors combined with anti-PDL1 antibody*** | Glesatinib (MGCD265) | Carcinoma, Non-Small-Cell Lung | Completed | NCT02954991 | II | 2016 |
|  | Sitravatinib |  |  |  |  |  |
|  | Mocetinostat |  |  |  |  |  |
|  | Nivolumab |  |  |  |  |  |
|  | Bemcentinib (BGB324) | Melanoma | Recruiting | NCT02872259 | I | 2016 |
|  | Pembrolizumab |  |  |  | II |  |
|  | Dabrafenib/Trametinib |  |  |  |  |  |
|  | Bemcentinib (BGB324) | Lung Cancer Metastatic | Recruiting | NCT03184571 | II | 2017 |
|  | Pembrolizumab | NSCLC Stage IV |  |  |  |  |
|  |  | Adenocarcinoma of Lung |  |  |  |  |
|  | Bemcentinib (BGB324) | Lung Cancer Metastatic | Recruiting | NCT03184571 | II | 2017 |
|  | Pembrolizumab | NSCLC Stage IV |  |  |  |  |
|  |  | Adenocarcinoma of Lung |  |  |  |  |
|  | Bemcentinib (BGB324) | Mesothelioma, Malignant | Recruiting | NCT03654833 | II | 2018 |
|  | Rucaparib |  |  |  |  |  |
|  | Abemaciclib |  |  |  |  |  |
|  | Pembrolizumab |  |  |  |  |  |
|  | Atezolizumab |  |  |  |  |  |
|  | Bevacizumab |  |  |  |  |  |
|  | Dostarlimab |  |  |  |  |  |
|  | Niraparib |  |  |  |  |  |
|  | Sitravatinib (MGCD516) | Non-Small Cell Lung Cancer (NSCLC) | Recruiting | NCT04921358 | III | 2021 |
|  | Tislelizumab |  |  |  |  |  |
|  | Sitravatinib (MGCD516) | Carcinoma, Non-Small-Cell Lung | Recruiting | NCT04925986 | II | 2021 |
|  | Pembrolizumab | Lung Diseases |  |  |  |  |
|  |  | Lung Neoplasms |  |  |  |  |
|  |  | Metastatic Lung Non-Small Cell Carcinoma |  |  |  |  |
|  |  | Stage IV Lung Non-Small Cell Cancer AJCC v7 |  |  |  |  |
| ***Antibody-based dual therapy*** | Nivolumab | Renal Cell Carcinoma | Active | NCT03141177 | III | 2017 |
|  | Cabozantinib |  | Not recruiting |  |  |  |
|  | Sunitinib |  |  |  |  |  |
|  | Ipilimumab |  |  |  |  |  |
| ***Antibody*** | HuMax-AXL-ADC | Ovarian Cancer | Completed | Completed | I | 2017 |
|  | Enapotamab Vedotin | Cervical Cancer | Results not published |  | II |  |
|  |  | Endometrial Cancer |  |  |  |  |
|  |  | Non-Small Cell Lung Cancer |  |  |  |  |
|  |  | Thyroid Cancer |  |  |  |  |
|  |  | Melanoma |  |  |  |  |
|  |  | Sarcoma |  |  |  |  |
|  | BA3011 | Solid Tumor | Recruiting | NCT03425279 | I | 2018 |
|  | Mecbotamab vedotin | Non-Small Cell Lung Cancer |  |  | II |  |
|  |  | Melanoma |  |  |  |  |
|  |  | Sarcoma |  |  |  |  |
|  |  | Sarcoma, Ewing |  |  |  |  |
|  |  | Osteosarcoma |  |  |  |  |
|  |  | Leiomyosarcoma |  |  |  |  |
|  |  | Synovial Sarcoma |  |  |  |  |
|  |  | Liposarcoma |  |  |  |  |
|  |  | Soft Tissue Sarcoma |  |  |  |  |
|  |  | Bone Sarcoma |  |  |  |  |
|  |  | Refractory Sarcoma |  |  |  |  |
|  | BA3011 | Solid Tumor | Recruiting | NCT03425279 | I | 2018 |
|  | CAB-AXL-ADC | Non-Small Cell Lung Cancer |  |  | II |  |
|  |  | Melanoma |  |  |  |  |
|  |  | Sarcoma |  |  |  |  |
|  |  | Sarcoma, Ewing |  |  |  |  |
|  |  | Osteosarcoma |  |  |  |  |
|  |  | Leiomyosarcoma |  |  |  |  |
|  |  | Synovial Sarcoma |  |  |  |  |
|  |  | Liposarcoma |  |  |  |  |
|  |  | Soft Tissue Sarcoma |  |  |  |  |
|  |  | Bone Sarcoma |  |  |  |  |
|  |  | Refractory Sarcoma |  |  |  |  |
|  | Tilvestamab (BGB149) | Healthy Volunteers | Completed | NCT03795142 | I | 2019 |
|  | CAB-AXL-ADC (BA3011) | Non-Small-Cell Lung Cancer | Recruiting | NCT04681131 | II | 2020 |
| ***GAS-6 binding protein*** | Batiraxcept (AVB-S6-500) | Ovarian Cancer | Active | NCT03639246 | I | 2018 |
|  |  |  | Not recruiting |  |  |  |
| ***GAS-6 binding protein combined with anti-PDL1 antibody*** | Batiraxcept | Urothelial Carcinoma | Active | NCT04004442 | I | 2019 |
|  | (AVB-S6-500) |  | Not recruiting |  |  |  |
|  | Avelumab |  |  |  |  |  |
|  | Durvalumab | Platinum-Resistant Fallopian Tube Carcinoma | Active | NCT04019288 | I | 2019 |
|  | Batiraxcept | Platinum-Resistant Ovarian Carcinoma | Not recruiting |  | II |  |
| ***CAR T-based therapy*** | CCT301-38 | Renal Cell Carcinoma | Active | NCT03393936 | I | 2018 |
|  | CCT301-59 |  | Not recruiting |  | II |  |
